# Induction of Natural Defenses in Tomato Seedlings by Using Alginate and Oligoalginates Derivatives Extracted from Moroccan Brown Algae

**DOI:** 10.3390/md18100521

**Published:** 2020-10-19

**Authors:** Meriem Aitouguinane, Soukaina Bouissil, Anouar Mouhoub, Halima Rchid, Imen Fendri, Slim Abdelkafi, Mohamed Didi Ould El-Hadj, Zakaria Boual, Pascal Dubessay, Christine Gardarin, Philippe Michaud, Zainab El Alaoui-Talibi, Cherkaoui El Modafar, Guillaume Pierre, Cédric Delattre

**Affiliations:** 1 Laboratoire Agrobiotechnologie et Bioingénierie, Faculté des Sciences et Techniques Guéliz, Université Cadi Ayyad, Marrakech 40000, Morocco; meriem.aitouguinane@etu.uca.fr (M.A.); soukaina.BOUISSIL@etu.uca.fr (S.B.); anouar.mouhoub@edu.uca.ac.ma (A.M.); zainab.elalaouitalibi@gmail.com (Z.E.A.-T.); elmodafar@uca.ac.ma (C.E.M.); 2 CNRS, SIGMA Clermont, Institut Pascal, Université Clermont Auvergne, F-63000 Clermont-Ferrand, France; pascal.dubessay@uca.fr (P.D.); christine.gardarin@uca.fr (C.G.); philippe.michaud@uca.fr (P.M.); guillaume.pierre@uca.fr (G.P.); 3 Laboratoire de Biotechnologies et Valorisation des Ressources Végétales, Faculté des Sciences, Université Chouaib Doukkali, El Jadida 24000, Morocco; rchidhalima@hotmail.com; 4 Laboratoire de Biotechnologies des Plantes Appliquées à l’Amélioration des Plantes, Faculté des Sciences, Université de Sfax, Sfax 3038, Tunisia; imen.fendri@fss.usf.tn; 5 Laboratoire de Génie Enzymatique et Microbiologie, Equipe de Biotechnologie des Algues, Ecole Nationale d’Ingénieurs de Sfax, Université de Sfax, Sfax 3038, Tunisia; slim.abdelkafi@enis.tn; 6 Laboratoire de Protection des Ecosystèmes en Zones arides et Semi-Arides, Ouargla Université, Université Kasdi Merbah, Ouargla 30000, Algeria; mohameddidi@yahoo.fr (M.D.O.E.-H.); Biozakaria1983@gmail.com (Z.B.); 7 Institut Universitaire de France (IUF), 1 rue Descartes, 75005 Paris, France

**Keywords:** marine seaweed, alginate, oligoalginates, elicitation, PAL activity, phenolic compounds, tomato seedling

## Abstract

Polysaccharides extracted from marine algae have attracted much attention due to their biotechnological applications, including therapeutics, cosmetics, and mainly in agriculture and horticulture as biostimulants, biofertilizers, and stimulators of the natural defenses of plants. This study aimed to evaluate the ability of alginate isolated from *Bifurcaria bifurcata* from the Moroccan coast and oligoalginates derivatives to stimulate the natural defenses of tomato seedlings. Elicitation was carried out by the internodal injection of bioelicitor solutions. The elicitor capacities were evaluated by monitoring the activity of phenylalanine ammonia-lyase (PAL) as well as polyphenols content in the leaves located above the elicitation site for 5 days. Alginate and oligoalginates treatments triggered plant defense responses, which showed their capacity to significantly induce the PAL activity and phenolic compounds accumulation in the leaves of tomato seedlings. Elicitation by alginates and oligoalginates showed an intensive induction of PAL activity, increasing from 12 h of treatment and remaining at high levels throughout the period of treatment. The amount of polyphenols in the leaves was increased rapidly and strongly from 12 h of elicitation by both saccharide solutions, representing peaks value after 24 h of application. Oligoalginates exhibited an effective elicitor capacity in polyphenols accumulation compared to alginate polymers. The alginate and oligosaccharides derivatives revealed a similar elicitor capacity in PAL activity whereas the accumulation of phenolic compounds showed a differential effect. Polysaccharides extracted from the brown seaweed *Bifurcaria bifurcate* and oligosaccharides derivatives induced significantly the phenylpropanoid metabolism in tomato seedlings. These results contribute to the valorization of marine biomass as a potential bioresource for plant protection against phytopathogens in the context of eco-sustainable green technology.

## 1. Introduction

Today, pesticides are among the most widely used substances in agriculture worldwide to control losses caused by phytopathogens. The use of these chemical compounds in crop protection has major impacts on the environment, and public health [1]. The increasing thrust toward the development of sustainable agriculture and green revolution technologies has led to the emergence of new molecules derived from natural sources, biopesticides. Despite their non-toxic mechanisms and eco-friendly manner, their usefulness is increasingly restricted because of their expensive cost of production, the broad spectrum, which might lead to negative effects on non-target organisms, and low reliability due to low stability [2]. In this context, new promising alternative approaches were developed by researchers, one of them, the stimulation of the natural plant defenses using natural elicitors that does not exert selective effects on the pathogen. This strategy aims to induce plant resistance against a broad spectrum of microorganisms [3].

Seaweed offers an abundant and valuable source of bioactive constituents, they have been recognized with their application in human food, nutraceutical, cosmetic, and pharmaceutical industries as well as agri-horticultural sectors, as fertilizers and soil conditioners [4]. Recently, oligo/polysaccharides, derived from green, red, and brown macroalgae have gained much attention among researchers due to their elicitor activities leading to stimulate plant defense mechanisms and protect against a wide range of phytopathogens [5]. Ulvan from green seaweed, laminarin isolated from brown algae as well as carrageenan from red ones, and their oligomers derivatives, are extensively studied due to their capacity to enhance various plants defenses [5,6,7,8,9,10]. In the case of alginate purified from brown seaweed and oligoalginates, only few studies have been conducted.

This study aimed to investigate the ability of alginate isolated from the brown seaweed *Bifurcaria bifurcata* and oligosaccharides derivatives obtained after radical depolymerisation, to induce the phenylpropanoid defense pathway, in particular, phenylalanine ammonia-lyase (PAL) activity and phenolic compounds accumulation in the leaves of tomato seedlings. This is the first report on these saccharides as elicitors of tomato plant defenses.

## 2. Results and Discussion

### 2.1. Structural and Chemical Characterization of the Alginate Extracted from Bifurcaria bifurcata and Their Oligomers Derivatives

Alginate was extracted from the brown seaweed *Bifurcaria bifurcata* from the Moroccan coast. The polysaccharides were extracted through a series of steps involving acid and alkali heat treatment and purified by rinsing several times with ethanol, and then analyzed chemically and structurally [11,12]. Oligoalginates were obtained after radical depolymerization of the alginate by aqueous hydrogen peroxide (H_2_O_2_) solution for 6 h at 70 °C. The hydrolysis process was described in detail in the previous study [11]. Extraction and purification techniques were potentially optimized to achieve higher yield of target compound. As described in the previous study [12], the yield of alginate was about 24% (*w/w*), and the chemical composition was revealed as 48.6% of total sugars, with the abundance of uronic acids (58.4%), and the presence of minor impurities (phenolic compounds, proteins, and sulfate groups). Total carbohydrates were determined by the Dubois method [13]. In general, two colorimetric methods can be used, the Dubois and the anthrone assays [14]. Both methods give different results depending on the standard sugars used and the composition of carbohydrates in the biological samples. These colorimetric assays remain problematic and could involve an over or underestimation of carbohydrate content, because of the presence of various biochemical compounds that can have a possible interaction in the quantification [15]. Extracted alginate showed substantial quantitative yields as well as an appropriate composition, to make it a good product for biological activities. Widely, the alginate yield of studied brown seaweed genus, such as *Sargassum*, *Turbinaria* [16], *Cystoseira* [17,18], *Laminaria* [19] is largely in the range of 20–50% (*w/w*). The reported alginate yield and chemical properties depend greatly on the algal species and its polysaccharide content, harvesting conditions, and extraction methods [20]. Naturally, algae live in a very changing environment, which leads to morphological, content, and composition diversity. Several studies have already shown temporal and spatial variability in the content and composition of algae compounds when exposed to various environmental factors, such as nutrients, temperature, salinity, or light [21]. Furthermore, the season of seaweed cultivation also plays an important role in their structural and biochemical composition [22].

In order to predict the different mannuronic and guluronic acid residues ratio (M/G ratio) of intact alginate powder and to determine the block structure, ^1^H-NMR spectroscopy was performed. The molecular weight was determined by the High-Performance Steric Exclusion Chromatography HPSEC. Alginate from brown seaweed *Bifurcaria bifurcata* was characterized by a weight average molecular weight (Mw) of 22.5 × 10^4^ g/mol, and 5500 g/mol for the produced oligoalginates. The estimated M/G ratio from ^1^H-NMR spectra shows higher values of guluronic acid (68%) than mannuronic acid blocks (32%, M/G < 1), indicating that the samples are rich in guluronic acid. All structural and chemical analyses of alginate were presented in the previous study with a detailed description [12]. The determination of the composition of alginates and their physical properties is an important criterion for knowing the gelling properties in order to select the appropriate concentrations for biological tests. In general, alginates with an M/G ratio (<1) form strong and brittle gels, making them suitable for biomedical or environmental applications, whereas alginates with M/G ratio (>1) produce an elastic gel that can be used for many industrial applications [23]. In this work, the concentration used for elicitation was 0.3% (*w/v*), which means that the flow behavior of the sodium alginate has a Newtonian effect. This avoids several irregular modifications of the functions and cellular mechanisms due to the high viscosity of the solution. Previous studies have emphasized that lower concentrations (<2% *w/v*) of alginates in aqueous solution represent Newtonian flow while, higher concentrations (>2% *w/v*) improve viscosity and shear thinning behavior [24,25,26,27]. The bioelicitor concentration of 3 g/L was chosen according to the previous study, which was carried out under the same conditions on tomato seedlings [6].

### 2.2. Polysaccharides from Bifurcaria bifurcata and Derived Oligosaccharides Induce Defense Responses in Tomato Seedlings

#### 2.2.1. Effect of Alginate Extracted from *Bifurcaria bifurcata* and Oligoalginates Derivatives on the PAL Activity

The induction of the phenylpropanoid defense pathway in the leaves located above the elicitation area of the tomato seedlings in response to alginates, and oligoalginates treatments was evaluated by monitoring the PAL activity and the phenolic compounds biosynthesis over 5 days. The phenylpropanoid mechanisms play an important role in the signaling pathways of plant defense in response to phytopathogens attack as well as bioelicitor application [28]. This pathway is activated by the formation of cinnamic acid via the action of phenylalanine ammonia-lyase, the key enzyme in the defense system that initiates both primary (shikimate pathway) and secondary (phenylpropanoid) metabolisms [29].

**Figure 1:**
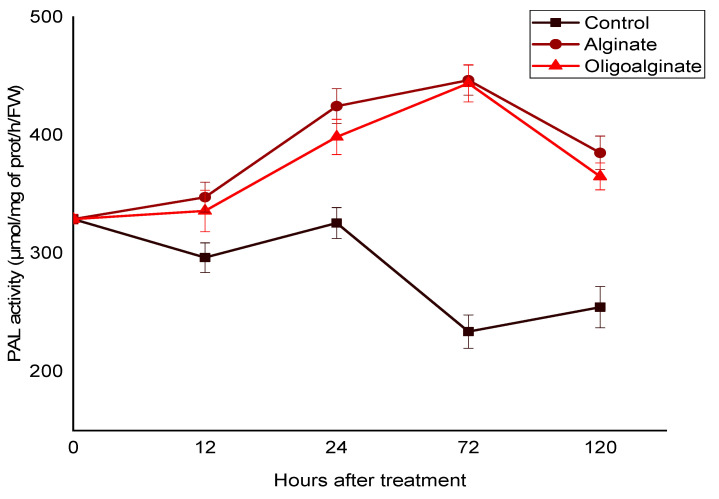
Effects of alginate and oligoalginates derivatives on PAL activity in the leaves located above the elicitation site of tomato seedlings for 5 days. Each value is the mean of four repetitions ± SE.

The application of alginate and oligoalginates derivatives (3 g/L), by internodal infiltration in the tomato seedlings induced significantly the PAL activity in the leaves (*p* < 0.05). The elicitation response depended on the treatment duration (Figure 1). The PAL activity increased substantially in the leaves for each of the alginate and their oligomers (*p* < 0.05) and remained at high levels throughout the incubation period. In the response to alginate treatment, levels rose steadily from 12 h of elicitation, reaching a peak value 2-fold higher compared to control (water) after 72 h of treatment, to be decreased slightly in remaining days. Besides, the elicitation by oligoalginates derivatives showed a similar pattern. As illustrated in Figure 1, both alginate and oligoalginates exhibited similar effectiveness and efficiency to enhance PAL activity in the leaves of tomato seedlings.

#### 2.2.2. Effect of Alginate Extracted from *Bifurcaria bifurcata* and Oligoalginates Derivatives on the Phenolic Compounds Content

**Figure 2:**
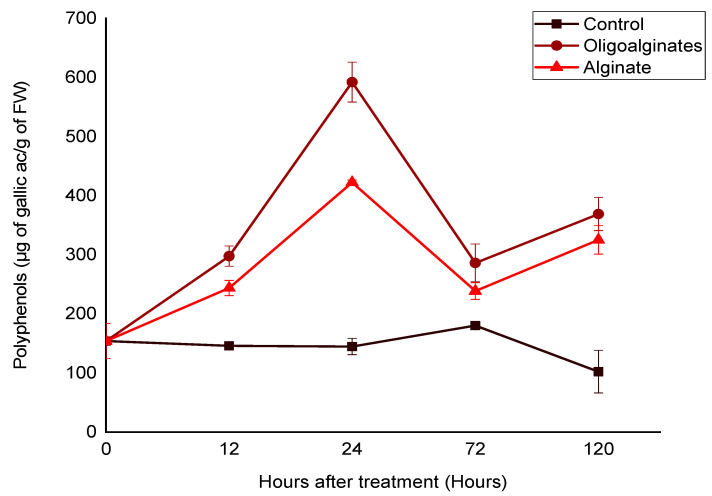
Effects of alginate and oligoalginates derivatives on phenolic contents in the leaves located above the elicitation site of tomato seedlings for 5 days. Each value is the mean of four repetitions ± SE.

The phenolic compounds synthesis in the leaves of tomato seedlings was significantly promoted (*p* < 0.05), in response to treatment with alginate and oligoalginates derivatives (3 g/L). As illustrated in Figure 2, the polyphenol content was enhanced by both bioelicitor applications compared to the control (water). The elicitation response depended on the saccharide elicitor and the duration of treatment. For each of alginate and their oligosaccharides derivatives, the accumulation of these metabolites in the leaves showed similar trends, it initially increased rapidly and strongly from 12 h of elicitation, representing peaks value after 24 h of application. This maximal level was 2.6 and 4 times higher as compared to untreated plants (control), for alginate and oligoalginates, respectively. Thereafter, levels suddenly decreased attaining a control value after 72 h of treatment to increase significantly in the remaining days. Compared to alginate, oligoalginates were more effective, they expressed an intense elicitor capacity in polyphenols synthesis, that was almost 1.5 times more important than that observed in response to alginate polymer after 24 h of treatment (Figure 2).

These studies revealed clearly, the capacity of alginates, extracted from the brown seaweed *Bifurcaria bifurcata*, and oligoalginates derivatives to induce PAL activity (Figure 1) and polyphenols biosynthesis (Figure 2) in the leaves of tomato seedlings. Based on these results, and on previous study realized on date palms using the same alginates [12], we suggest that polysaccharides isolated from *Bifurcaria bifurcata* and their oligomers could be important elicitors to trigger the natural defenses of different plants enhancing protection against a wide range of phytopathogens. Additional studies are therefore required for exploring the protective effects of these molecules. As far as we know, very limited research has examined the stimulator activities of alginate and oligoalginates purified from brown algae. However, other polysaccharides extracted from green and red algae and their oligomers have been extensively studied for their potential to enhance a wide range of defense reactions in different plants, such as ulvan, the sulphated polysaccharide from green seaweeds [6,7,30,31], carrageenan from red seaweed [9,32,33,34], laminarin and fucoidan from brown seaweed [35,36,37,38,39].

Alginate and oligoalginates derivatives expressed an important and persistent elicitor effect of PAL activity (Figure 1). This was supplemented by a strong accumulation of phenolic compounds in the leaves of tomato seedlings, in the response of both saccharide bioelicitors. However, oligosaccharides presented an effective elicitor capacity compared to alginate polymers (Figure 2). Similar contributions were observed in a recent study, carried out on tomato plants after application of ulvan, oligoulvans, glucuronan, and oligoglucuronans from green seaweed [6]. Treatment with these saccharide elicitors increased significantly the PAL activity as well as the content of phenolic compound in the leaves above and below the elicitation site, this response was systemic and seemed to be related to the salicylic acid pathway [6]. The application of these saccharide molecules reduced significantly wilt development caused by *Fusarium oxysporum* f. sp. *lycopersici*, particularly the oligoulvans which strongly reduced wilting and mortality of tomato plants, by 44% and 54%, respectively, compared to the control plants [6]. The results presented by Abouraïcha et al. reported that the PAL activity and phenolics content have been greatly enhanced in the apple fruit after elicitation by ulvan and oligoulvans, indicating the effectiveness of these saccharides in protection against blue and grey molds [7]. The implication of the PAL activity and polyphenols on plant resistance after seaweed polysaccharides treatment have been largely reported [7,8,40,41].

On a worldwide scale, tomato constitutes an essential vegetable fresh crop. In spite of its economic importance, bacterial and fungal diseases and pests became the major common constraints accounting for poor tomato production [42]. Bacterial disease caused by *Xanthomonas campestris* pv. *vesicatoria*, early blight (*Alternaria linariae*), late blight (*Phytophthora infestans*), and powdery mildew (*Golovinomyces lycopersici*) are considered the most disastrous problems of tomato [43,44]. For instance, several reports have shown that the application of crude or purified algal preparations protect against a large range of pathogens in tomato seedlings [42,45,46].

The results revealed a similar elicitor capacity in PAL activity whereas the accumulation of phenolic compounds showed a differential effect in the response of alginate and oligosaccharides derivatives. PAL is one of the most thoroughly studied enzymes, being the first committed enzyme that controls the interface between phenylalanine and the secondary phenylpropanoid metabolism in response to biotic and abiotic stress in plants [47]. After the first reaction, which consists of catalyzing the phenylalanine in the presence of PAL enzymes, polyphenols are produced by three different biogenetic routes, the shikimate, malonate, and mevalonate pathways. The shikimate pathway is the most important in the biosynthesis of the majority of plant phenolics which comprise a diverse group of molecules including flavonoids (flavones, anthocyanidins), stilbenes, tannins, lignans, and lignin [47]. These compounds play an important role in structural and defense-related functions. A recent study has revealed that high levels of these metabolites at the site of pathogen invasion can restrict or slow the growth of the pathogen [7]. The shikimic acid pathway consists of many sequential enzymatic steps, each reaction produces different metabolites that play an important role as branch point compounds serving as factors that induce the synthesis of various phenolic compounds in the plant [48]. Seven enzymes could intervene in this pathway for the development of the majority of secondary metabolites depending on the elicitor molecules [47,48,49]. From this, it can be suggested that the difference in the expression of phenolic compounds in the leaves of tomato seedlings between alginates and oligoalginates could be explained by the distinct target of the enzymatic reactions stimulated by each elicitor in the shikimate pathway. The presence of phenolic compounds synthesized with polyphenol oxidase and peroxidase enzymes in plants may also influence the content of these metabolites.

Additionally, the deferential elicitor responses between alginates and their oligomers might be associated to the saccharide chain length as well as the structure of both oligosaccharides and polysaccharides, which leads to multiple variations in cell surface interaction which means widely varying receptors that are important for recognition, regulation, and signal transduction. In this work, the oligoalginates derivatives were characterized by 2 ≤ DP (degree of polymerization) ≤ 24. A series of recent studies have indicated that seaweed-derived oligosaccharides with a low degree of polymerization (DP) strongly stimulate plant defense mechanisms compared to polysaccharides. Tomato seedlings and apple fruit treated with oligoulvans and oligoglucuronans, obtained after enzymatic depolymerization from ulvan and glucuronan, respectively, revealed a strong induction of PAL activity and other defense-related enzymes and metabolites, that were more effective than polysaccharides [6,7,40]. In previous studies, it was suggested, that oligoalginates enriched polymannuronic acid (Poly-M) fraction, obtained after chemical depolymerization, induced a sustained increase in PAL activity in wheat and tobacco leaves, while, polyguluronic acid (Poly-G) showed low elicitor activity, mentioning that only Poly-M activate defense responses in plants probably through the salicylic acid (SA) signaling pathway [50,51]. However, other studies reported that some metabolites could be induced by high DP instead of low DP [35], although all of them could act as elicitors. It has been demonstrated, in tobacco, that although two different oligosaccharides have similar DP, defense responses varied from each other [52]. Compared with curdlan oligosaccharides with low DP, laminarin showed a durable effect on plant defense mechanisms, whereas oligomers took an active part in early defense reactions [52]. Chitin is a polymer of *N*-acetyl-D-glucosamine, found in the cell walls of fungi. Barber et al. reported that the monomer and dimer of this molecule were inactive, while the tetramer, pentamer, and hexamer significantly induced lignin synthesis in wheat leaves [53]. On the other hand, the smaller chitooligosaccharides (COSs) with DP between 3 and 6 showed lower elicitor activity. In contrast, pentameric and hexameric COSs (DP ≥ 20) effectively stimulated cellular responses in rice [54]. In addition, the production methods of oligosaccharides from native polymers, including chemical hydrolysis or enzymatic hydrolysis, could influence the elicitor behavior of these molecules. A previous study conducted on animal cells showed that enzymatically depolymerized alginate oligomers (unsaturated guluronate and mannuronate oligomers) were highly active triggers for cytokine production, while saturated guluronate and mannuronate oligomers produced by acid hydrolysis were less effective. This suggests that the unsaturated terminal structure with double bond is important for the bioactivity of alginate oligomers, regardless of molecular size or structure [55]. This hypothesis has yet to be proven on plant cells.

The difference between elicitors effectiveness could also depend on the specificity of the receptors in the cell wall towards these molecules, which activate the defense signaling pathways in different ways. Plants have a perceptual system that is responsible for regulating the defense against foreign molecules, called elicitors, which can be from pathogens (fungi, bacteria, viruses, insects, etc.), animals, plants, or seaweeds [56]. These general elicitors are designated as PAMPs (pathogen-associated molecular patterns) when isolated from pathogenic agents, MAMPs (Microbe-Associated Molecular Patterns) from non-pathogenic microorganisms and chemical compounds or DAMPs (Damage-Associated Molecular Patterns) corresponding to endogenous signals derived from the plant host resulting from the action of pathogens [57]. They are recognized by surface-based receptors called PRRs (pattern recognition receptors) or by specific intracellular receptors that recognize the specific pathogenic effector molecules [58,59]. PRR surface receptors are known as plasma membrane resident proteins, including receptor-like kinases (RLK), receptor-like proteins (RLP), and extracellular binding proteins. In contrast, intracellular receptors, known as NB-LRR, constitute the nucleotide-binding site (NB) and leucine-rich repeat (LRR) [60].

Regarding the recognition of poly-oligosaccharides by plants, it has been shown that ^14^C-labeled mycolaminarin isolated from *Phytophthora* sp. presents a high-affinity binding site in membrane preparations from soybean roots [61]. Other studies have demonstrated the presence of a single class of specific binding receptors with high affinity for β-glucan oligosaccharide elicitor in the cell membrane preparations from the root and other parts of the soybean cotyledon [62]. A radio-labeled chitin oligosaccharide elicitor also showed a high-affinity binding site in the plasma membrane of rice [63], tomato cells [64], and soybean root [65]. Lectins and Lectin Receptor-Like Kinases seem to have an important role in signaling and perception cascades activating defense mechanisms in response to saccharide elicitors. It has been identified as carbohydrate-binding proteins [66]. Therefore, it is essential to understand the interaction between these elicitors and the host to develop durable resistance in plants and to find new approaches for disease control. For this, more specific studies are planned to investigate the mechanisms underlying the perception of polysaccharides and oligosaccharides derived from marine seaweed.

## 3. Materials and Methods

### 3.1. Collection of *Bifurcaria bifurcata* Seaweed Samples

The brown seaweed *Bifurcaria bifurcata* was collected from the intertidal zone at low tide, from the Moroccan Atlantic coast in the south of El Jadida city (Latitude 32°15′ to 33°15′; longitude 7°55′ to 9°15′), during December 2017. After harvesting, the samples were washed extensively with seawater, then transported to the laboratory in plastic bags and rinsed again with distilled water in order to discard salt, sand, and any foreign particles that could affect the biological activities. The seaweed biomass was air-dried in the dark for 15 days, oven-dried for 6 h at 50 °C, and then blended into a fine powder using an electric mill (Moulinex (a subsidiary of Groupe SEB, Lyon, France)).

### 3.2. Preparation of Sodium Alginate and Oligoalginates Derivatives Extracts

The polysaccharides and oligosaccharides used in this study were provided in the Institut Pascal, University Clermont Auvergne in France. The extraction and purification of alginate and oligoalginates derivatives, from the brown algae *Bifurcaria bifurcata*, were previously described in detail [11,12], and the Chemical Analysis, High-Performance Steric Exclusion Chromatography (HPSEC) (Agilent Technologies, Santa Clara, CA, USA) and NMR Spectroscopy Analysis (Bruker Scientific Instruments, Billerica, MA, USA), were established according to the published protocol.

### 3.3. Plant Cultivation and Elicitor Applications

**Figure 3:**
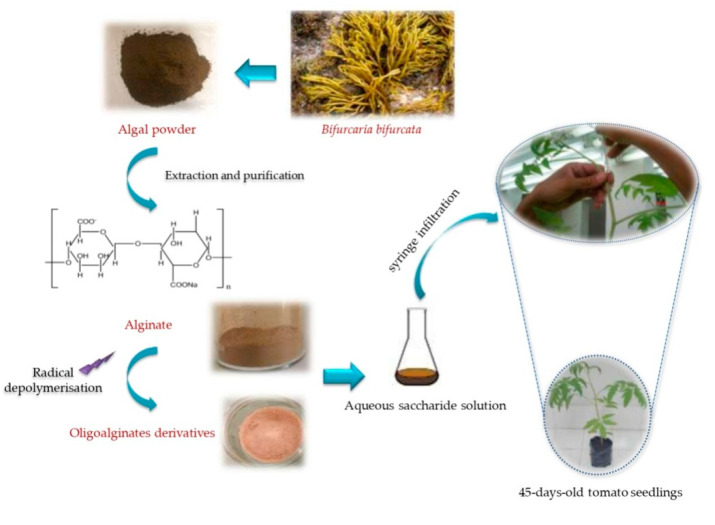
Overview of the elicitor application method.

Sterile Campbell 33 tomato seeds were used. The seeds were sown in individual 7 cm pots containing peat substrate for germination. Two weeks after growing, the seedlings were transplanted into plastic poly grow bags (22 × 10 × 8 cm) containing a mixture of soil and peat (*v/v*). The plants were grown in a culture room at 26 °C and a cycle of 16 h/8 h (light/dark). When the seedlings were 45 days old, the elicitation was carried out by syringe infiltration of 20 μL of each saccharide solution (3 g/L in distilled water, pH 6) into the internodal middle of the tomato seedlings. The control plants were injected with distilled water. The shoot system lengths of all plants used were 50 cm, at the eight-leaf stage. Eight tomato seedlings were collected after 0, 12, 24, 72, and 120 h in each treatment group. The two apical leaf samples from two plants were pooled, frozen in liquid nitrogen, and ground in a mortar and then stored at −20 °C for subsequent analysis. All experiments were conducted thrice under identical conditions. Figure 3 summarizes the elicitor application method.

### 3.4. Extraction and Measurement of Phenylalanine Ammonia Lyase (PAL) Activity

Tomato leaf powders were weighed and macerated in 2 mL of extraction buffer (100 mM Tris-HCl, pH: 7, 14 mM 2-mercaptoethanol, and 3% Polyvinylpolypyrrolidone (PVPP)). The homogenate was centrifuged (UNIVERSAL 320 R, Andreas Hettich GmbH & Co. KG, Tuttlingen, Germany) at 10,000× *g* for 30 min at 4 °C and the supernatant was collected. The enzyme reaction mixture contained 1 mL of 100 mM Tris-HCl, pH: 6, 200 µL of 20 Mm l-phenylalanine, and 200 µL of enzyme extract. The reaction was carried out at 30 °C for 30 min and stopped by addition of 100 µL of 6 N HCl [6]. The PAL activity was estimated by measuring *trans*-cinnamic acid synthesized at 290 nm using an extinction coefficient of 16.8901 L/mol/cm [67]. The PAL specific activity was expressed in µmole of *trans*-cinnamic acid/mg of protein/h/mg of fresh weight (FW).

### 3.5. Extraction and Quantification of Total Phenolics Contents

The leaves powder was weighed and extracted in 2 mL of acetone 80%. Samples were incubated in an ultrasonic bath for 30 min at 4 °C. The extracts were then centrifuged at 10,000× *g* for 15 min at 4 °C. Two hundred microliters of the acetonic extract was added to 250 µL of the Folin–Ciocalteau reagent (1/3) and 500 µL of sodium carbonate (20%). The samples were kept at 40 °C for 30 min [68]. The Folin–Ciocalteau (FC) assay shows interference from many oxidizable compounds such as tyrosine, tryptophan, ascorbic acid, and urea, and even diethyl ether [69]. To avoid the interaction of possible non-phenolic substances in the calorimetric estimation, another assay was performed under the same conditions by adding 3% of PVPP to the phenolic extract. After stirring and centrifugation, the obtained supernatant was treated according to the same procedure described above. The absorbance was measured at 760 nm (Jenway 6305 Spectrophotometer, Jenway, Dunmow, Essex, UK). The difference between the first (without PVPP) and the second measurement (with PVPP) was calculated to determine the amount of total phenolic compounds (gallic acid equivalent µg/g of FW).

### 3.6. Protein Activity Assays

Total soluble protein was measured by the Bradford method, using bovine serum albumin (BSA) as the standard [70]. One hundred microliters of each sample were added to 2 mL of diluted Bio-Rad protein assay reagent (1/5). After 10 min incubation in the dark at room temperature min, the absorbance was measured at 595 nm.

### 3.7. Data Analysis

The data for the comparison of the PAL activity and the accumulation of phenolic compounds in the leaves of tomato seedlings between treated and control plants in each period of elicitation were analyzed using ANOVA test in SPSS version 25.0 software (IBM SPSS Statistics for Windows, Armonk, NY). The results were presented as mean value ± standard error (SE) of four replicates with two tomato seedlings per replicate. Statistical differences were considered significant at *p* < 0.05.

## 4. Conclusions

In this study, it appears likely that the alginates isolated from the brown seaweed *Bifurcaria bifurcata* and oligosaccharides derivatives possessed elicitor activities. They showed the capacity to induce phenylpropanoid metabolism by enhancing the phenylalanine ammonia-lyase (PAL) activity and phenolic compounds accumulation in the leaves of tomato seedlings. The oligosalginates presented an effective elicitor capacity compared to alginate polymers. Additional studies are planned to understand more completely this difference in the mechanisms of interaction between polysaccharides and oligosaccharides derived from marine seaweed and the plants upon the expression of marker genes and transcriptomic analysis. Further studies are also needed to ensure the protective effects of these molecules against phytopathogens. Therefore, these results obtained highlight molecules of marine algae likely to contribute to the resistance of plants to pathogens by reducing/replacing the use of synthetic pesticides.
